# Automated segmentation of haematoma and perihaematomal oedema in MRI of acute spontaneous intracerebral haemorrhage

**DOI:** 10.1016/j.compbiomed.2019.01.022

**Published:** 2019-03

**Authors:** Stefan Pszczolkowski, Zhe K. Law, Rebecca G. Gallagher, Dewen Meng, David J. Swienton, Paul S. Morgan, Philip M. Bath, Nikola Sprigg, Rob A. Dineen

**Affiliations:** a Stroke Trials Unit, Division of Clinical Neuroscience, University of Nottingham, UK; b Radiological Sciences, Division of Clinical Neuroscience, University of Nottingham, UK; c Department of Medicine, National University of Malaysia, Malaysia; d Department of Neuroradiology, Nottingham University Hospitals, Queen's Medical Centre, Nottingham, UK; e Imaging Department, Leicester Royal Infirmary, Leicester, UK; f Department of Radiology, Royal Derby Hospital, Derby, UK; g Medical Physics and Clinical Engineering, Nottingham University Hospitals, Queen's Medical Centre, Nottingham, UK; h NIHR Nottingham BRC, UK

**Keywords:** Brain MRI, Image segmentation, Spontaneous intracerebral haemorrhage, Stroke

## Abstract

**Background:**

Spontaneous intracerebral haemorrhage (SICH) is a common condition with high morbidity and mortality. Segmentation of haematoma and perihaematoma oedema on medical images provides quantitative outcome measures for clinical trials and may provide important markers of prognosis in people with SICH.

**Methods:**

We take advantage of improved contrast seen on magnetic resonance (MR) images of patients with acute and early subacute SICH and introduce an automated algorithm for haematoma and oedema segmentation from these images. To our knowledge, there is no previously proposed segmentation technique for SICH that utilises MR images directly. The method is based on shape and intensity analysis for haematoma segmentation and voxel-wise dynamic thresholding of hyper-intensities for oedema segmentation.

**Results:**

Using Dice scores to measure segmentation overlaps between labellings yielded by the proposed algorithm and five different expert raters on 18 patients, we observe that our technique achieves overlap scores that are very similar to those obtained by pairwise expert rater comparison. A further comparison between the proposed method and a state-of-the-art Deep Learning segmentation on a separate set of 32 manually annotated subjects confirms the proposed method can achieve comparable results with very mild computational burden and in a completely training-free and unsupervised way.

**Conclusion:**

Our technique can be a computationally light and effective way to automatically delineate haematoma and oedema extent directly from MR images. Thus, with increasing use of MR images clinically after intracerebral haemorrhage this technique has the potential to inform clinical practice in the future.

## Highlights

• A fast method for detection of haematoma and oedema in haemorrhagic stroke is presented.
• To our knowledge, it is the first method to use magnetic resonance images only.
• The introduced method is fully automated, training-free and unsupervised.
• Comparable results with respect to human expert annotations are achieved.
• Method has potential to inform clinical practice, with increased clinical MRI use.

## 1 Introduction

Spontaneous intracerebral haemorrhage (SICH), the commonest cause of haemorrhagic stroke, is a devastating condition associated with 1-year survival rates of 46% [1] and high rates of disability in survivors [2]. This type of haemorrhagic stroke is caused by rupture of a vessel within the brain parenchyma and subsequent extravasation of blood into surrounding tissues. This results in a haematoma with variable amounts of oedema in adjacent brain tissue.

Haematoma volume and oedema are both clinically important as they are independent predictors of outcome after ICH [[3], [4], [5], [6]]. Therefore, providing a method for segmentation of haematoma and perihaematoma oedema in the acute and early subacute phase (between 2 and 7 days from the onset of symptoms) using Magnetic Resonance (MR) data is of clinical relevance, as measurement of the volume and diffusion characteristics of oedema surrounding haematoma provides a potential tool to evaluate the inflammatory response around SICH [[7], [8], [9]]. Additionally, the identification of haematoma location and size provides a seed for oedema localisation and serves as a baseline for longitudinal measurement of haematoma expansion. Furthermore, for application in large clinical trials it is critical that such segmentation method is fully automated, since manual delineation of haematoma and oedema is extremely time-consuming and, as such, not routinely performed. Hence, we introduce an automated method for segmentation of haemorrhage and perihaematoma oedema to assist in the evaluation of SICH patients recruited to both the TICH-2 study [10,11] and its nested MRI sub-study [12].

Previous work on haematoma and/or oedema segmentation in SICH has been mainly focused on processing CT images. One of the earliest approaches is the one proposed by Loncaric et al. [13], where a method based on k-means histogram-based clustering for haematoma, and on iterative morphological processing for oedema segmentation is presented. Majcenic and Loncaric [14] propose a stochastic method based on simulated annealing for segmentation of CT images depicting SICH. The work by Pérez et al. [15] proposes two semi-automated SICH segmentation methods. The first one is based on 3D morphology operations, while the second one combines live wire and graphical information retrieval techniques. A semi-automated approach that combines a region growing technique to segment haematoma and a level-set method to delineate oedema in CT is presented by Bardera et al. [16]. The work by Zaki et al. [17] introduces a multi-level local segmentation method based on fuzzy c-means clustering for haematoma detection. A modified distance regularised level set evolution algorithm for haematoma segmentation is proposed in the paper by Bhanu Prakash et al. [18]. In the study by Bhadauria et al. [19], the authors present a segmentation approach which combines fuzzy c-means and a region-based active contour technique. An oedema extraction approach based on region growing is presented by Chen et al. [20]. The seeds for the region growing algorithm are obtained using the expectation-maximisation clustering method. Zhang et al. [21] introduces an approach for intracerebral and intra-ventricular haemorrhage detection on CT using adaptive thresholding based on local contrast in different window sizes and case-based reasoning. Finally, Volbers et al. [22] propose to use a fixed threshold range of between 5 and 33 Hounsfield units in CT for oedema delineation. They further show that the resulting segmentations in these CT images have a strong correlation with manual ones performed on their MRI counterparts. Despite the simplicity of such approach, it is based on a general criterion which is not driven by the actual MR images and, thus, there may be cases where a slightly different range could be more appropriate. A drawback of CT-based methods is that, even though haemorrhages are in most cases very conspicuous, perihaematomal oedema delineation is a very challenging task [23]. Additionally, magnetic resonance T2-weighted images (including T2-weighted FLAIR) are generally considered superior to CT for quantification of perihaematomal oedema [24,25]. Moreover, some of them focus on haemorrhage segmentation only or oedema segmentation only.

As previously presented in a review [26], there is a substantial amount of work on segmentation of ischaemic stroke lesions from MR images [[24], [25], [26], [27], [28], [29], [30], [31], [32], [33], [34], [35], [36], [37], [38], [39], [40]]. In addition, there are other methods either not covered by such review or developed after its publication [[41], [42], [43], [44], [45], [46], [47], [48], [49], [50], [51]], and also efforts like the ISLES MICCAI challenge [52]. However, these methods are not directly applicable to unsupervised automated segmentation of haemorrhagic lesions from MR images. This is because they require training data and/or user interaction, require special types of MR acquisitions, or are tailored to the specific image appearances of ischaemic lesions.

This paper describes a revised and extended version of our previous preliminary work [53]. To our knowledge, no other previous work on automated unsupervised haematoma and/or oedema segmentation from MR images following SICH has been proposed. A possible exception to this might be the work of Wang et al. [54]. However, the authors propose a processing method that aids ICH segmentation by mapping tissue susceptibility property without blooming artefacts, rather than a segmentation method per se. The possible lack of focus on MR segmentation of intracerebral haemorrhages may be explained by the fact that CT is still the predominant modality for SICH care clinical trials. Moreover, intracerebral haemorrhage account for a comparatively small proportion of all strokes: 10% in high-income countries and 20% in low- and middle-income ones [55]. Finally, the window of time measured from onset on which a high contrast between haematoma, oedema and surrounding tissue can be clearly seen on MRI is relatively small (from around 12 h to approximately 6 days) [56]. With this in mind, we take advantage of the improved contrast seen on MR in the acute and early subacute phase to provide a reliable segmentation of haematoma and surrounding perihaematoma oedema using a training-free, unsupervised and fully automated method. We evaluate this method by comparing segmentation results against those performed by five different expert raters, and against *DeepMedic* [51], a state-of-the-art brain lesion segmentation approach. Our proposed technique was written in MATLAB (The MathWorks, Inc, MA) and the source code is made publicly available at https://github.com/stefanpsz/ICH_segmentation.

## 2 Materials and methods

### 2.1 SICH subjects

We use a dataset of MR images coming from 50 patients recruited to the TICH-2 MRI sub-study [12], all of whom had to be previously recruited into the main TICH-2 study [10,11]. Informed consent was obtained separately for each study, either from the patient itself or one of their relatives. On this set of patients, 44.0% are female, the mean age is 64.3 years (range 20–88 years), and the mean scanning time point from randomisation is 4.2 days (range 1.8–6.8 days). Patients were scanned in one of 16 different centres using scanners from various manufacturers and a range of acquisition parameters (see Supplementary Material).

### 2.2 Pre-processing of MR sequences

We utilise 2-dimensional axial T2* gradient recalled echo (GRE) and FLAIR sequences, and a 3-dimensional T1 sequence per subject. We also have T2 and diffusion-weighted images available, but they are not used in this work. T2 images are not used since we observe that all the information that they provide (such as oedema extent) is already present in the FLAIR images and with better contrast. Diffusion-weighted images are excluded as the TICH-2 MRI sub-study protocol specifies standard echo-planar sequences, which have large spatial distortions and registration-based correction is unreliable. We rigidly register the T2* GRE and FLAIR sequences to their corresponding T1 sequence using the Medical Image Registration ToolKit (MIRTK).^1^ When resampling the images, B-Spline interpolation is used as an effort to minimise undesirable partial volume averaging effects. From this point of the paper, any mention of T2* GRE and FLAIR images will refer to the T1-registered ones.

### 2.3 Image masks

In order to create image masks for each subject, a T1 template is non-linearly registered to the subjects’ T1 sequence after affine initialisation using MIRTK. We chose to utilise an age-specific template [57], since it is based on healthy individuals with ages similar to what is commonly seen in stroke and, hence, better suited for registration with SICH patients. We segment this template into 138 labels using the *MALP-EM* segmentation method [58] with Pincram brain extraction [59] enabled. We further modify the resulting 138-label map by setting all the unlabelled voxels within the Pincram brain mask to the “CSF” (cerebrospinal fluid) label (label 18).

#### 2.3.1 Subject brain mask

We transform the *MALP-EM* label map using the computed transformations between the age-specific template and each subject T1 sequence. We then binarise this map, yielding an initial whole-brain mask in subject space. We also create a T2* GRE and a FLAIR mask by removing all padded voxels (i.e., voxels with an intensity value of zero) in the corresponding sequence. We then perform *hole-filling-closing* of the mask with one iteration in both T2* GRE and FLAIR masks, so potential zero-valued voxels within the brain parenchyma are not left out. We define *hole-filling-closing* with N iterations as N morphological dilations with a kernel of 3 × 3 × 3 voxels, followed by hole-filling with 6-connected neighbourhood, and then by N morphological erosions with the same kernel. The final whole-brain mask is the intersection of all three masks followed by 2 morphological erosions.

#### 2.3.2 Susceptibility and lesion masks

The susceptibility mask is meant to delineate areas of the brain that are prone to present susceptibility artefacts in the T2* GRE sequence, and the lesion mask delineates subcortical areas where haemorrhages occur more commonly. Both masks are used later in our method and are created by combining a number of labels in the transformed subject space *MALP-EM* map that we identified as being located in the specified areas (see Table 1 and Table 2).

**Table 1:** MALP-EM labels considered as being prone to be located in areas that are sensitive to present susceptibility artefacts in the T2* GRE sequence.

Label	Name	Label	Name
10/11	Right/Left cerebellum exterior	75/76	Right/Left medial frontal cortex
45/46	Right/Left anterior orbital gyrus	81/82	Right/Left medial orbital gyrus
59/60	Right/Left frontal pole	89/90	Right/Left middle temporal gyrus
61/62	Right/Left fusiform gyrus	91/92	Right/Left occipital pole
63/64	Right/Left gyrus rectus	93/94	Right/Left occipital fusiform gyrus
65/66	Right/Left inferior occipital gyrus	111/112	Right/Left posterior orbital gyrus
67/68	Right/Left inferior temporal gyrus	119/120	Right/Left subcallosal area
71/72	Right/Left lateral orbital gyrus	133/134	Right/Left temporal pole

**Table 2:** MALP-EM labels considered as defining the subcortical areas where haemorrhages are more likely to occur.

Label	Name	Label	Name
3/4	Right/Left accumbens area	19/20	Right/Left hippocampus
7	Brain stem	25/26	Right/Left pallidum
8/9	Right/Left caudate	27/28	Right/Left putamen
12/13	Right/Left cerebellum white matter	29/30	Right/Left thalamus proper
16/17	Right/Left cerebral white matter		

#### 2.3.3 Ventricle and white-grey matter masks

Two separate masks are also generated. One is a mask including all ventricular labels in the transformed subject space *MALP-EM* map (see Table 3). The second mask includes only the white matter (WM) and grey matter (GM) areas and is obtained by simply removing from the whole-brain mask all voxels which coincide with label 18 (CSF) or with any of the ventricular labels.

**Table 3:** Ventricular labels in MALP-EM maps.

Label	Name
1	3rd ventricle
2	4th ventricle
21/22	Right/Left inferior lateral ventricle
23/24	Right/Left lateral ventricle

**Fig. 1:**
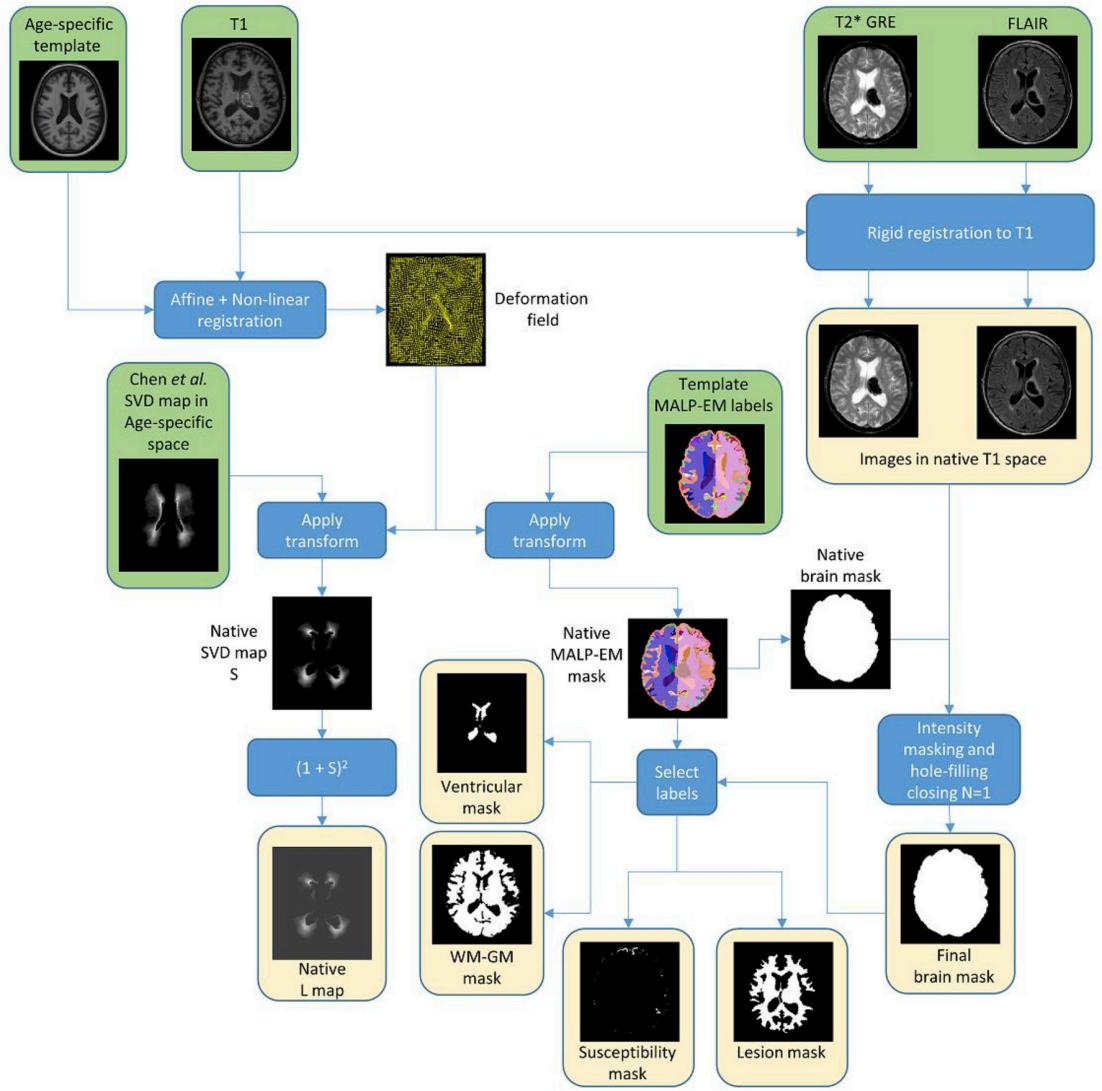
Pre-processing workflow including registration and generation of the different brain masks used in the proposed method. Green boxes correspond to inputs and yellow boxes correspond to outputs.

The entire pre-processing workflow, including intra- and inter-subject registrations and generation of the different brains mask is described in Fig. 1.

### 2.4 Haematoma segmentation

**Fig. 2:**
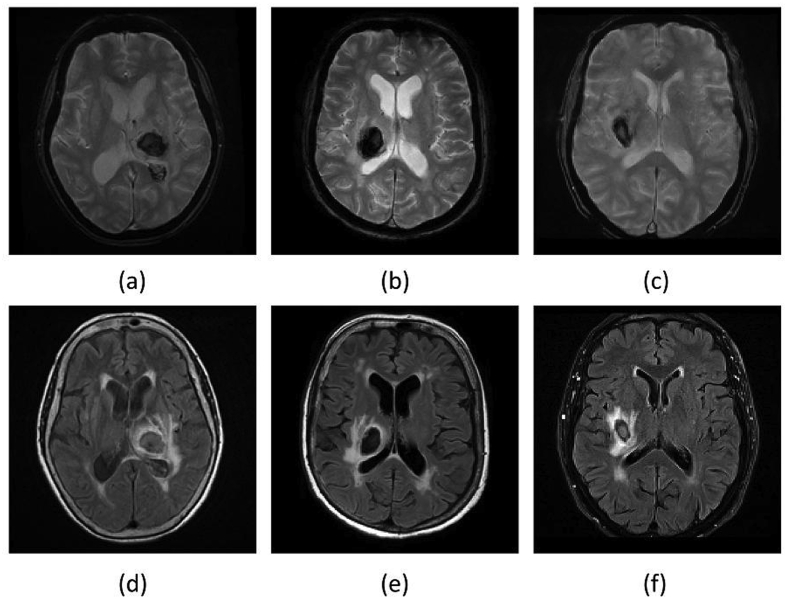
Late subacute phase MR sequences of three different SICH patients. (a)–(c): T2* GRE. (d)–(f): FLAIR. Note that on FLAIR imaging the haematoma can be completely iso-intense to grey matter, completely hypo-intense, or hypo-intense with an iso-intense centre. On the contrary, the haematomas tend to consistently appear hypo-intense in the T2* GRE images, albeit with a possible small iso-intense centre.

For haematoma segmentation, we base on the observation that, during the acute and early subacute phase, these regions appear mostly hypo-intense in T2* GRE. They may also appear hypo-intense with an iso-intense centre. However, they can be hypo- or iso-intense in FLAIR (see Fig. 2). We also observe that haematomas have adjacent hyper-intensities in FLAIR corresponding to surrounding oedema, with a high contrast between the two in the acute and early subacute phase [56]. Taking this into account, we propose to find the hypo-intense voxels in the T2* GRE image and hyper-intense voxels in FLAIR by considering them as outliers. To detect these outliers, we compute a robust intensity mean and standard deviation within the brain mask independently on the T2* GRE and FLAIR sequences using the Minimum Covariance Determinant (MCD) estimator [60,61]. We follow the chi-squared-based robust outlier estimation threshold given by Hubert and Debruyne [62], and only consider voxels that lie within the WM-GM mask. This is because this is where haemorrhages are more likely to occur, and because white matter and grey matter tend to be iso-intense in both T2* GRE and FLAIR, hence any hypo- or hyper-intensity is a potential outlier.

We now have a voxel-wise binary map of T2* GRE hypo-intensities $M^{hypo}$. We also have a voxel-wise binary map of FLAIR hyper-intensities $M^{hyper}$. In order to find the haematoma location, one could simply consider $M^{hypo}$ as the set of haematoma voxels. However, susceptibility artefacts in T2* GRE may produce hypo-intense regions away from the haematoma, and also make the extent of haematoma to appear larger than it actually is, making this assumption invalid. In consequence, we propose to look for the 6-neighbourhood connected components $C_{i}^{T2*}$ of the T2* GRE hypo-intense map and find the one which satisfies:

(1) $$C_{best}^{T2*}=\arg\underset{i}{\max}\;w_{i}\cdot S\left(C_{i}^{T2*}\right)$$

(2) $${w_{i}=\left(o_{i}\right)}^{2}\cdot \sqrt{\frac{l_{i}+1}{s_{i}+1}}$$

(3) $$S\left(x\right)=\left|x\right|\cdot {\left(\frac{\left|x\right|}{\left|B\left(x\right)\right|}\right)}^{2}=\;\frac{|x{|}^{3}}{{\left|B\left(x\right)\right|}^{2}}$$

Here, $o_{i}=\left|C_{i}^{T2*}\cap M^{hyper}\right|$ is the number of voxels in the connected component that overlap with the consolidated hyper-intensity map, and $s_{i}$ and $l_{i}$ correspond to the number of voxels in the connected component $C_{i}^{T2*}$that are part of the susceptibility and lesion masks described in section 2.3.2, respectively. In Equation (3), B(x) is the minimal bounding box containingx, and |x| corresponds to the volume (number of voxels) ofx. We regard $w_{i}$ as a weighting term that favours components potentially surrounded by a large extent of hyper-intensities (i.e. oedema), which have a large proportion of its voxels located in subcortical areas identified as having high likelihood of presenting haemorrhages (via the lesion mask). At the same time, we penalise components coming from areas of potential susceptibility artefacts. Note also that S(x) corresponds to the area multiplied by the square of the extent. We consider the latter as a surrogate measure of solidity and roundness.

As mentioned before, susceptibility artefacts in T2* GRE often make the haematoma to appear larger than it actually is. This is not the case in FLAIR, where the haematoma is depicted in its true size. Therefore, we now focus on the FLAIR intensities in voxels belonging to $C_{best}^{T2*}$. The idea is that FLAIR voxels in this region will mainly include hypo- or iso-intense voxels belonging to the haematoma, and some hyper-intense voxels corresponding to oedema (due to the aforementioned susceptibility artefacts). Therefore, we propose to only consider as potential haemorrhage voxels those which belong to $C_{best}^{T2*}$ and have FLAIR intensities which are less than a haemorrhage threshold H computed as:

(4) $$H=\;\{\begin{array}{cc} \mu & if\;\mu \geq \nu \\ \;\;\mu +6\left(\mu -\nu \right) & otherwise \end{array}$$

**Fig. 3:**
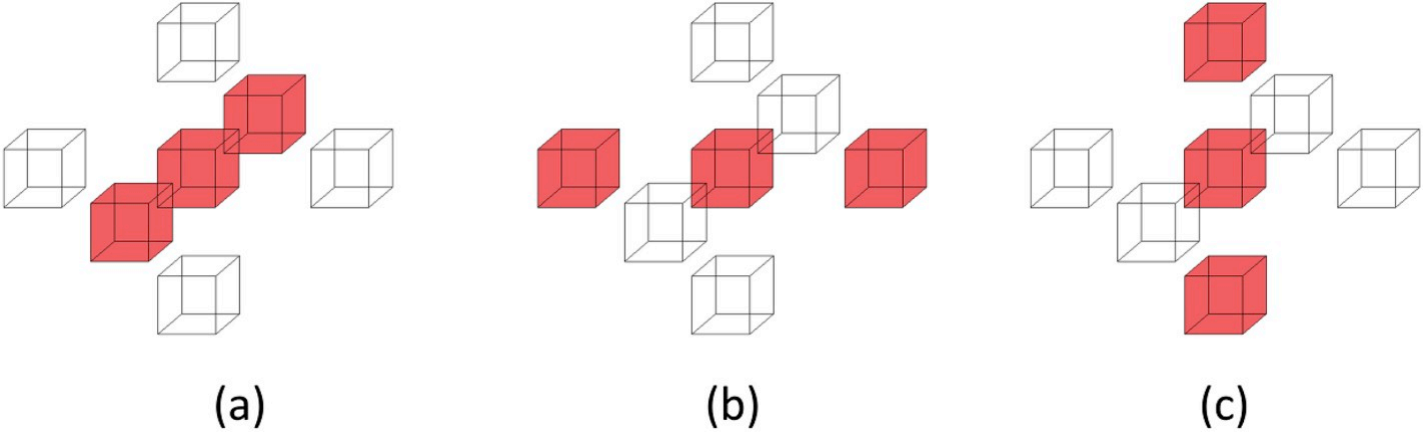
Linear patterns for detecting weak connections. We set the centre voxel to false if its 26-connected neighbourhood forms the (a), (b) or (c) pattern. The 20 neighbours that are always false in all three patterns are not depicted for ease of visualisation.

Here, μ and ν are the mean and median FLAIR intensity in $C_{best}^{T2*}$, respectively. The idea behind the computation of H is that $C_{best}^{T2*}$ should contain mostly haemorrhage voxels and some oedema, hence the mean intensity is a good estimate of a threshold. However, $C_{best}^{T2*}$ can potentially include a substantial amount of normal tissue due to susceptibility artefacts in the T2* GRE image, skewing the mean towards higher intensity values (negative skewness, i.e. μ<ν). Therefore, we correct the threshold value in that case to have a value lower than μ which is at 2k standard deviations σ from it, where $k=\frac{3\left(\mu -\upsilon \right)}{\sigma }\;$is the Pearson's second skewness coefficient. After computing the binary FLAIR haemorrhage voxels map by thresholding using H, we separate potential *weakly-connected* components by detecting voxels in the map for which their 26-connected neighbourhood forms a linear pattern (see Fig. 3) and setting them to false. Then, we compute the resulting *strongly-connected* components $C_{i}^{FLAIR}$ and select the one that satisfies:

(5) $$C_{best}^{FLAIR}=\arg\underset{i}{\max}\;S\left(C_{i}^{FLAIR}\right)$$

**Fig. 4:**
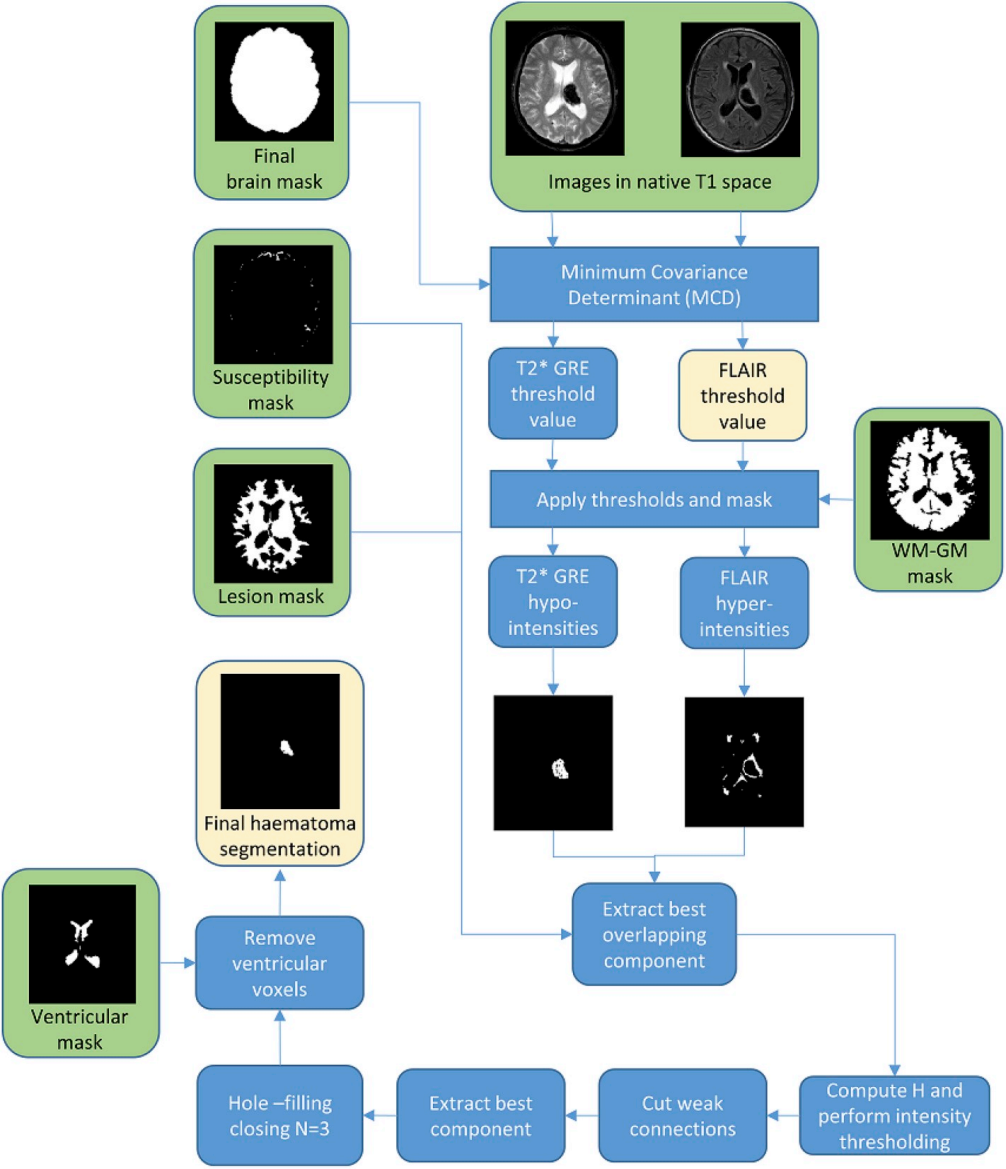
Haematoma segmentation workflow. Green boxes correspond to inputs and yellow boxes correspond to outputs.

The final haematoma segmentation is $C_{best}^{FLAIR}$, followed by *hole-filling-closing* with 3 iterations and removal of all voxels within the ventricle mask. The idea behind performing *hole-filling-closing* is to fill out potential areas of FLAIR iso-intensity within the haematoma which are greater than H. See Fig. 4 for a graphical description of the haematoma segmentation workflow.

### 2.5 Perihaematoma oedema segmentation

**Fig. 5:**
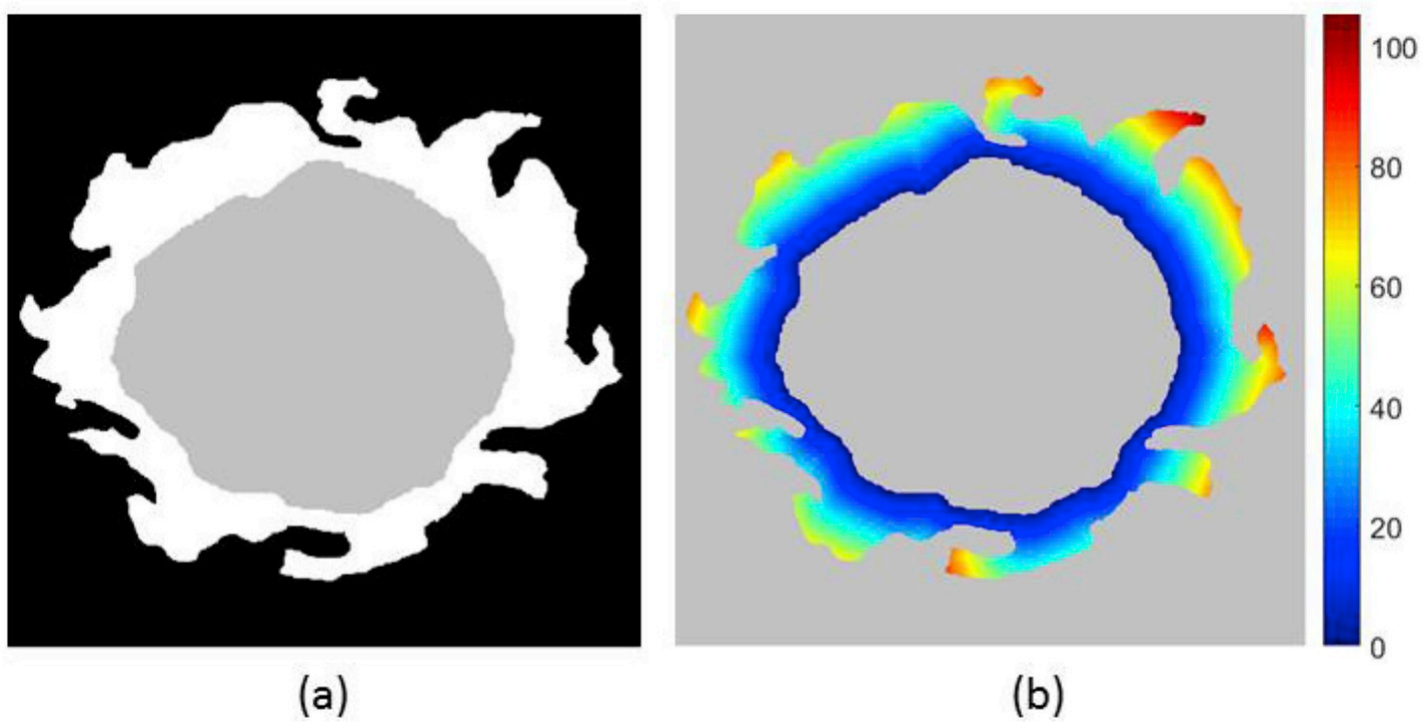
Visualisation of geodesic distance. (a) Simulated example of haematoma (grey) and hyper-intensity (white) segmentation. (b) Geodesic quasi-euclidean distance map from haematoma for each voxel belonging to hyper-intensity. Note that the distance is the shortest separation measured along the hyper-intense segmentation.

Once the haematoma segmentation is complete, we proceed to segment the oedema surrounding it. We consider oedema as occurring anywhere in the brain. Hence, the first step is to compute a *whole-brain* mask of FLAIR hyper-intensities using the FLAIR threshold value T previously computed by means of the MCD estimator. Then, one could simply regard this mask as the oedema region. However, voxels in this map may also be associated with presence of leukoaraiosis (white matter lesions). To alleviate this issue, we look to restrict possible oedema voxels to the ones that are close to the haematoma and are located in regions of low probability of leukoaraiosis. In order to accomplish this goal, we first compute the geodesic quasi-euclidean distance from every voxel in the FLAIR hyper-intensity map to the haematoma, using the algorithm described by Soille [63] (see Fig. 5). This yields a voxel-wise geodesic distance map D with values of infinity for every hyper-intense voxel not reachable from the haematoma. Secondly, a voxel-wise white matter lesion weight map L is calculated by using the transformation between the Age-specific T1 template and the subject's T1 sequence to propagate a small vessel disease (SVD) probability map constructed from 277 manually annotated cases, as presented by Chen et al. [64]. This yields a native space SVD probability map S. We then set L as:

(6) $$L=\;{\left(1+\;S\right)}^{2}$$

Thirdly, a voxel-wise hyper-intensity threshold map T that takes into account both the distance to haematoma and leukoaraiosis weight is computed as:

(7) $$T=T\;\cdot \;\frac{\left(D\cdot L\right)+\lambda }{2\lambda },$$

**Fig. 6:**
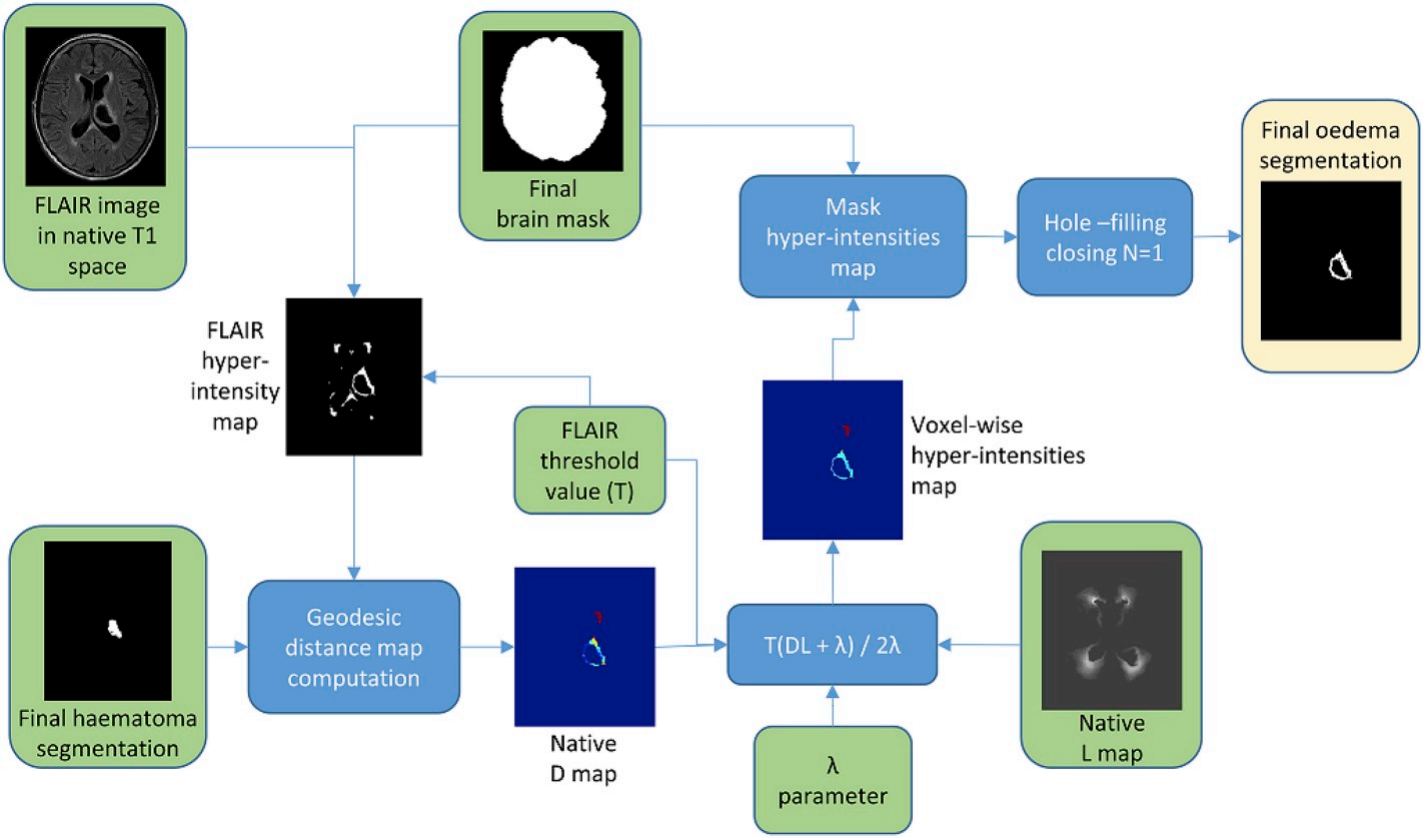
Perihaematoma oedema segmentation workflow. Green boxes correspond to inputs and yellow boxes correspond to outputs.

whereT is the FLAIR threshold value previously computed using the MCD estimator, λ>0 is a scalar parameter that controls the steepness of T around the haematoma region, and every operation is performed voxel-wise. We define an initial perihaematoma oedema segmentation as all voxels in the whole-brain mask which have a FLAIR intensity greater than T at the corresponding location. Finally, *hole-filling-closing* with one iteration is performed on this last segmentation to obtain the final result. We perform less iterations that in the haematoma case because the shape of oedema is much more irregular (e.g., dents in the surface might be artificially filled out with too many morphological operations). Fig. 6 shows the proposed oedema segmentation workflow.

### 2.6 Manual annotations and segmentation overlap

In order to be able to evaluate the performance of the proposed algorithm, manual annotations of haematoma and perihaematoma oedema were performed by 5 expert raters using ITK-SNAP^2^ [65] on a subset of 18 patients, which we refer to as Dataset A. The remaining 32 patients, which we refer as Dataset B, were annotated by rater 2 only with the same software. Please refer to the supplementary material for a description of the expertise of each rater. On Dataset A, 61.1% are female, the mean age is 65.8 years (range 35–86 years), and the mean scanning time point from randomisation is 4.2 days (range 1.8–6.2 days). On Dataset B, 34.4% are female, the mean age is 63.4 years (range 20–88 years), and the mean scanning time point from randomisation is 4.3 days (range 1.8–6.8 days).

We use the Dice score [66] as a measure of the segmentation agreement. This measure has a value of 0 for no overlap and a value of 1 for perfect overlap and is defined as:

(8) $$DS=\;\frac{2\;\cdot |X\;\cap Y|}{\left|X\right|+|Y|},$$

where X and Y are the two labellings being compared.

#### 2.6.1 Image registration details and algorithm parameterisation

All rigid intra-subject registrations were performed using Normalised Mutual Information (NMI) [67] with 64 histogram bins as similarity measure. The non-linear registrations of the age-specific T1 template to T1 subject sequences used a cosine similarity measure based on normalised gradient fields (cosNGF) [68], a transformation model based on B-spline free-form deformations [69], an image pyramid of 4 levels, bending energy (BE) as regularisation term, and 5 mm final control point spacing. The energy term weight distribution was set to 0.995 cosNGF +0.005 BE. The cosine similarity measure that we use is designed to be much less sensitive than standard similarities to missing correspondences [68], such as those introduced by the presence of haematoma and oedema.

Due to the subject images having a much larger field of view than the T1 template, we found that the best results are obtained by running similarity-then-affine registrations (i.e. 7 DOF then 12 DOF) of the T1 sequences to the T1 template with Local Cross Correlation [70] as similarity measure and a 5-level image pyramid, to then initialise the non-linear registrations using the inverse of the resulting transformation matrices.

The fraction of outliers the MCD algorithm should resist was set to be 40%. Additionally, we use λ=15 for automatic oedema segmentation in all cases, as we found this to be the optimal value (see Supplementary Material for details).

## 3 Results

### 3.1 Multiple-rater evaluation

**Fig. 7:**
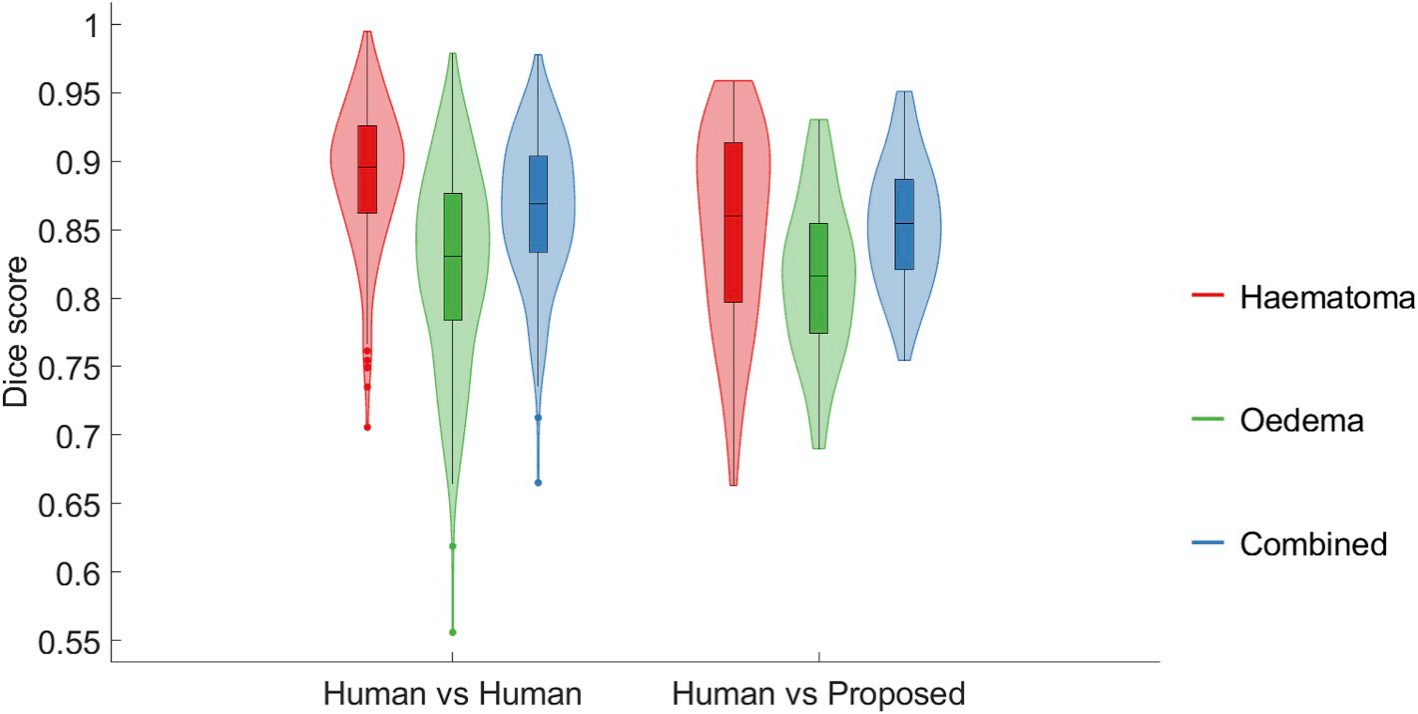
Box-and-violin plot of aggregated pairwise Dice overlaps between all 5 raters (Human vs Human) and aggregated Dice overlaps between the proposed algorithm and every expert rater (Human vs Proposed).

We study the pairwise overlap results between all 5 raters and the overlap results of the proposed method over Dataset A. Separate results for haematoma segmentation, perihaematoma oedema segmentation, and segmentation of the area spanned by the combination of both are presented. The results are shown in Fig. 7. Firstly, we draw the conclusion that correctly segmenting oedema tends to be more difficult than correctly segmenting haematoma, both for human raters and the proposed algorithm. This is expressed by a lower median score and greater variability in the oedema case. Secondly, we observe that the discrepancy between the distribution Dice scores achieved by the proposed algorithm and the distribution of those achieved by the human raters is statistically significant for ICH and combined labels (p<0.05 using Wilcoxon rank-sum testing), but not for oedema (p=0.141). Nevertheless, the scores attained by our method fall fully within the range of inter-rater scores. Lastly, we detect that Dice scores for the combined haematoma-oedema area tend to lie between those achieved for both labels individually. This may be explained by two main sources of errors: the incorrect delineation of the outer border of oedema, and incorrect delineation of the boundary between haematoma and oedema. These sources of errors could potentially be solved by employing a variational approach. However, this would require the introduction of an additional term in the energy function to account for the boundary of oedema and leukoaraiosis and more free parameters, which would require manual tuning.

**Fig. 8:**
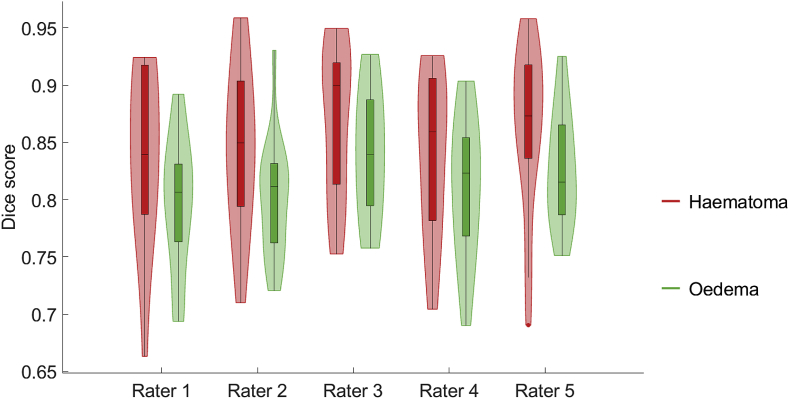
Box-and-violin plot of Dice overlaps for the proposed automated segmentation with respect to each rater over all 18 patients.

We also investigate the performance of our approach for each rater individually. The distribution of scores is depicted in Fig. 8. It is possible to observe that the proposed approach is consistently better at segmenting haematoma than at segmenting oedema. We also detect that the obtained Dice scores for both haematoma and oedema are not substantially different across raters.

**Fig. 9:**
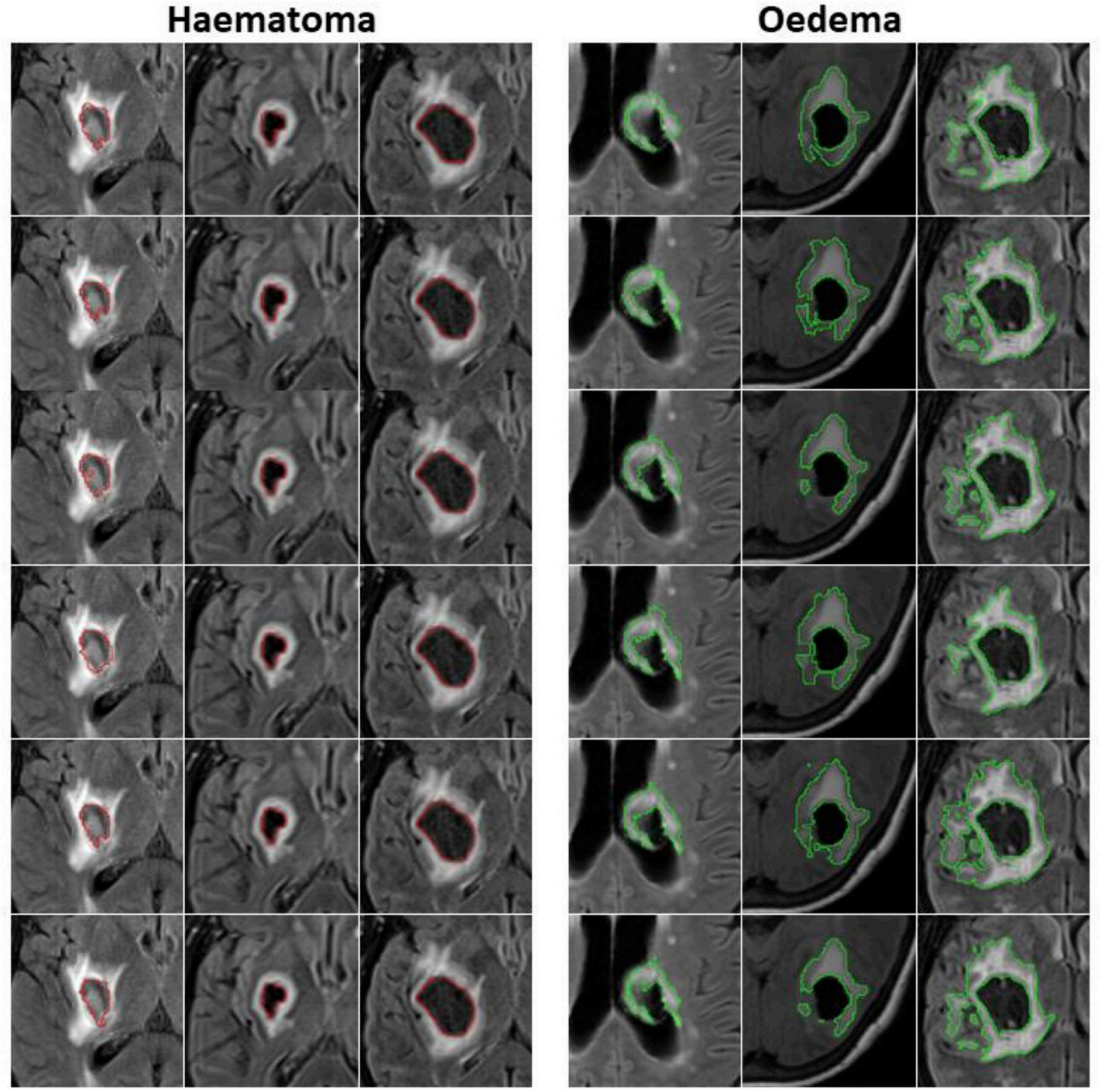
Visualisation of haematoma and perihaematoma oedema (λ=15) segmentation on Dataset A, overlaid on the FLAIR image. Worst case (left column), median case (middle column) and best case (right column), considering the average Dice score over all raters. Manual segmentations in rater order from top to bottom, with the bottom row being the proposed automated segmentation. Best viewed in colour.

**Fig. 10:**
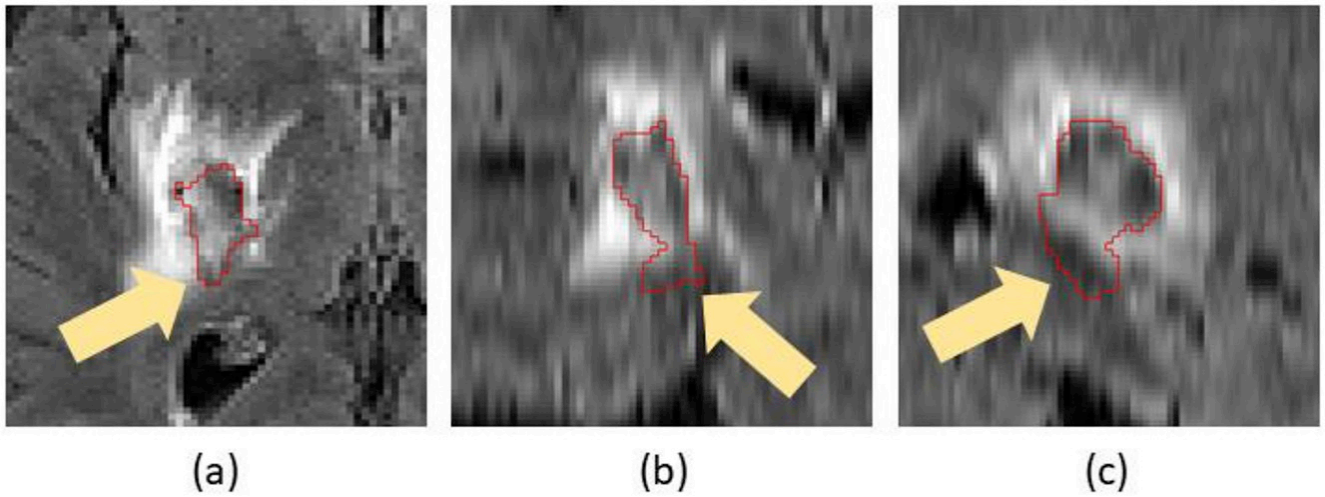
Worst case of haematoma segmentation on Dataset A. (a) FLAIR, axial. (b) FLAIR, coronal. (c) FLAIR, sagittal. Note that there is over-segmentation (yellow arrows) due to attenuated FLAIR intensities within oedema.

Additionally, visual results for patients in which the average Dice score over all raters is the worst, at the median,^3^ and the best are shown in Fig. 9. Note the proposed algorithm tends to overestimate the extent of haemorrhage in the worst case. This is mainly due to the attenuation of some FLAIR intensities within the oedema, which allows T2* GRE hypo-intensities associated with $C_{best}^{T2*}$ to “leak” (see Fig. 10). However, very good results are observed in the median and best cases, with mean Dice scores of 0.850 and 0.940, respectively. For oedema segmentation, a partial volume effect can be readily seen in the worst case, where the proposed method fails to segment a large area of oedema due to attenuated FLAIR intensities in the vicinity of CSF. Additionally, we observe that raters tend to have fairly different estimations of oedema labelling in the same case, negatively affecting the overlap evaluation. On the other hand, good overlaps are observed for the median and best cases, with mean Dice scores of 0.809 and 0.895, respectively.

### 3.2 Evaluation with state-of-the-art method

**Fig. 11:**
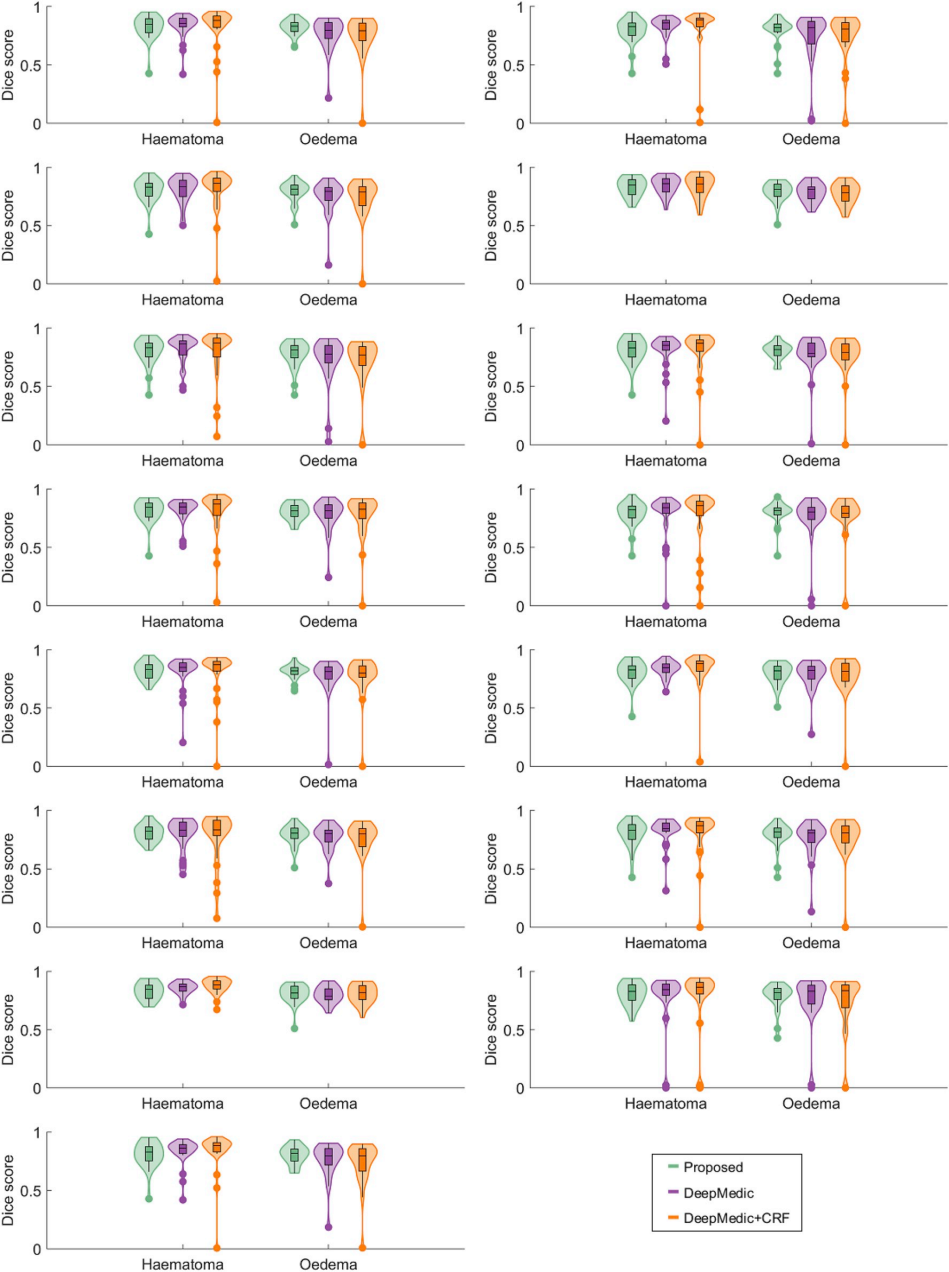
Box-and-violin plot of Dice overlaps for DeepMedic vs the proposed automated segmentation using random half-partitions of datasets A and B for training and testing.

We compare the performance of the proposed technique against a state-of-the-art MRI lesion segmentation method based on Fully Convolutional Neural Networks (FCNN), namely *DeepMedic* [51]. The *DeepMedic* FCNN is trained and tested using the pre-normalised T2* GRE and FLAIR images on Datasets A and B as input channels. Majority-voting label fusion amongst the five raters is used as consensus training ground truth for Dataset A, and the manual annotations of rater 2 for Dataset B. We randomly split Dataset A in two halves and Dataset B in two halves. One half from each dataset were combined to form the training data, and the remaining halves were combined to form the testing data. As the splitting of datasets involves randomness, we have repeated this experiment 15 times with different splitting. The resulting distributions for the proposed method and DeepMedic with and without Conditional Random Field post-processing are shown in Fig. 11. A slightly better performance can be observed for the proposed approach, although the differences are not statistically significant in any of the 15 cases (p>0.1 for both ICH and oedema using Wilcoxon rank-sum testing).

**Fig. 12:**
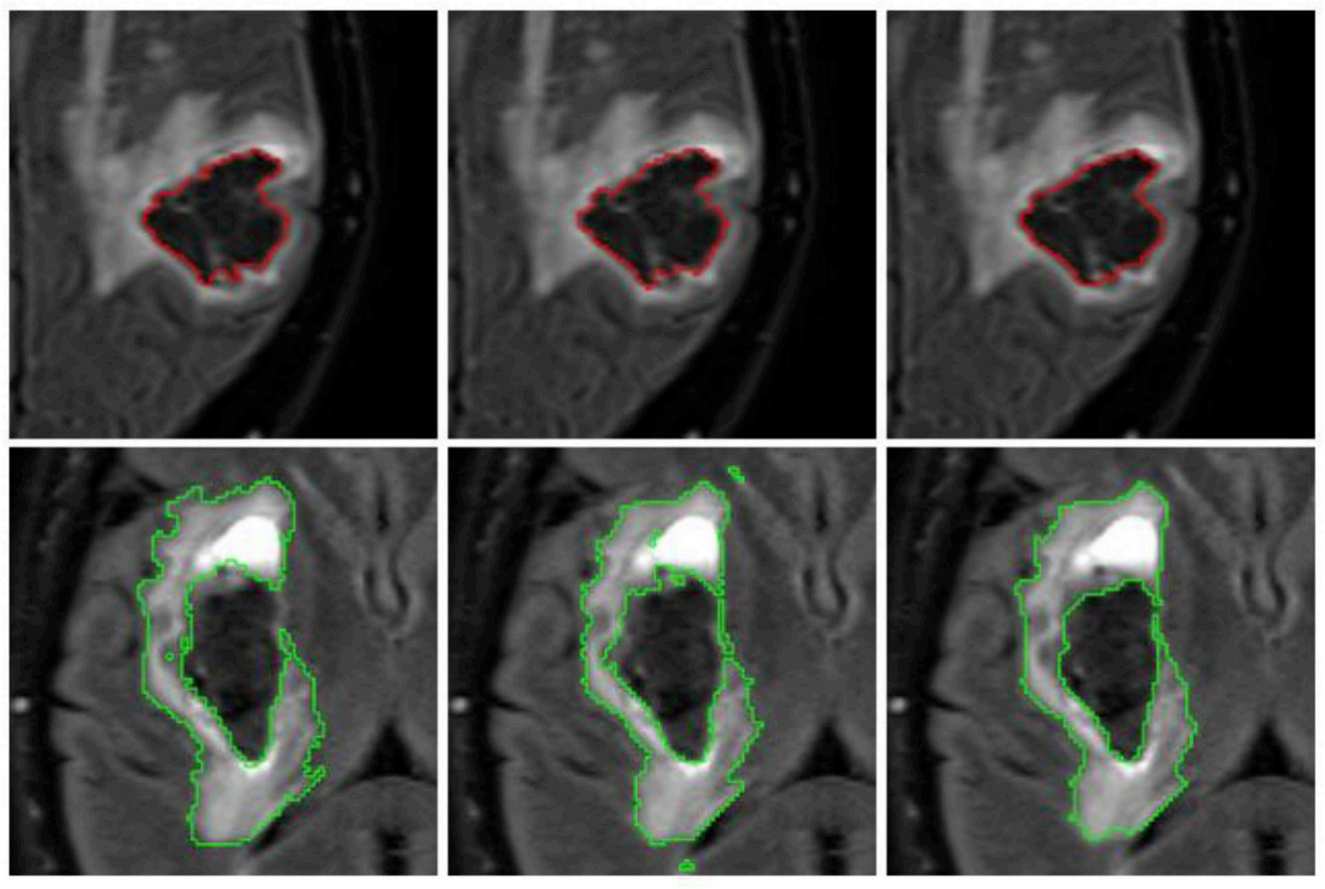
Visual results showing two Dataset B cases where a similarly good performance between the proposed method and DeepMedic is observed for both haematoma (top) and oedema (bottom). Left column: Manual segmentation. Middle column: DeepMedic segmentation. Right column: Proposed method.

Finally, visual haematoma and oedema segmentation results for Dataset B subjects in which both the proposed algorithm and *DeepMedic* perform well are depicted in Fig. 12. Additional visual results for some challenging subjects on the same dataset are provided in the Supplementary Material.

### 3.3 Influence of T1 registration in the segmentation

**Fig. 13:**
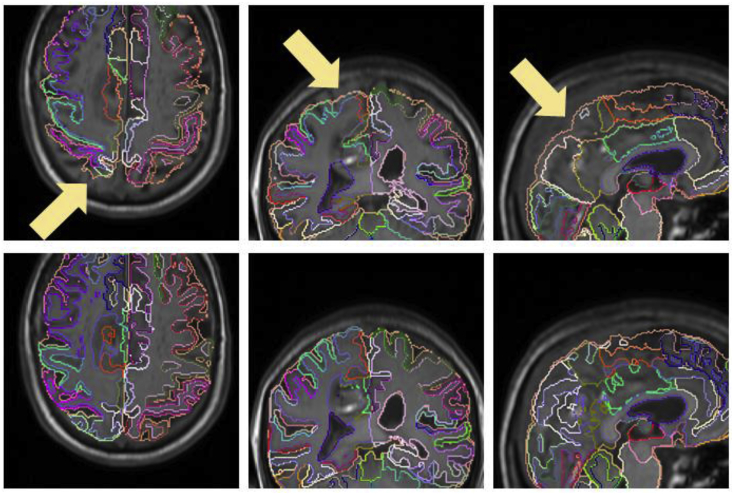
Axial, coronal and sagittal views of the proposed registration-based (top row) and re-labelled MALP-EM (bottom row) label maps of case 5, overlaid on the T1 image. We observe that re-labelled MALP-EM label map has a larger extent (see arrows in registration-based map for comparison), which resulted in a “leakage” of the T2* GRE region of interest and subsequent erroneous localisation of haematoma.

**Fig. 14:**
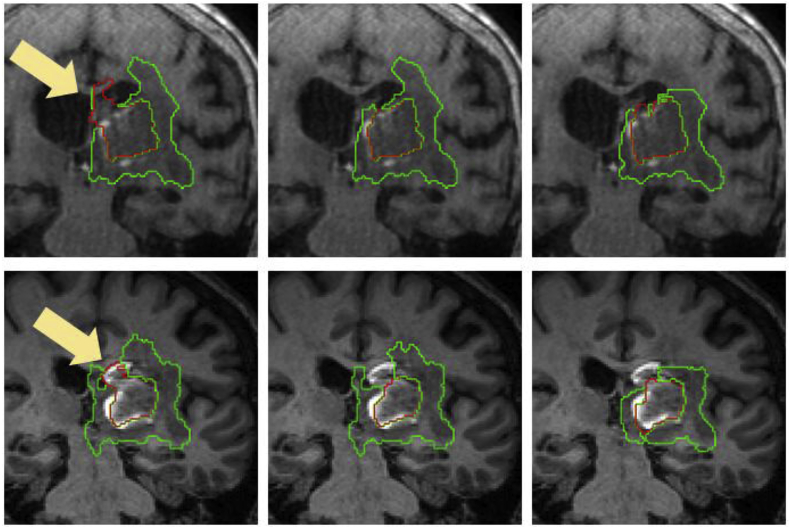
Segmentations overlaid on the T1 image for case 1 (top row) and case 2 (bottom row). Left column corresponds to the segmentation using the re-labelled MALP-EM map, middle column corresponds to the segmentation using the proposed registration-based map, and right column corresponds to the majority-vote label fusion amongst the five expert raters. Note that the re-labelled MALP-EM maps have non-ventricular labels for voxels corresponding to intraventricular haemorrhage, causing haematoma over-segmentation (arrows).

**Fig. 15:**
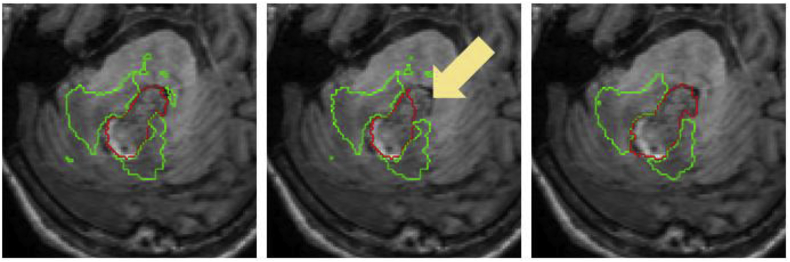
Segmentations overlaid on the T1 image for case 4. Left column corresponds to the segmentation using the re-labelled MALP-EM map, middle column corresponds to the segmentation using the proposed registration-based map, and right column corresponds to the majority-vote label fusion amongst the five expert raters. Note that the proposed registration-based label map incorrectly labels part of the haematoma as being within the fourth ventricle, resulting in under-segmentation of haematoma (arrow).

Since the *MALP-EM* segmentations of each subject are obtained indirectly via T1 registration with the age-specific template, we look at how this registration influences the performance of the proposed algorithm. Ideally, we should make a comparison using a manually delineated “ground truth” label map for each subject. However, this can take several days per subject for an expert neuroradiologist and is thus not feasible. Therefore, we create a proxy for this “ground truth” label map by performing *MALP-EM* directly on the subject's T1 image. We chose 8 cases from Dataset A that either have a haematoma close to the ventricles (and hence close to the WM-GM mask border) or have large haematomas causing considerable spatial distortion. We subsequently re-label all the voxels matching haematoma or oedema in the majority-voting label fusion amongst the five raters with an additional “lesion” label, which we consider as part of the lesion mask described in Section 2.3.2. We then run the proposed algorithm twice for each of the 8 subjects, once using the proposed registration-based label map and once using the re-labelled *MALP-EM* map. We compare the segmentations using both types of label maps with the majority-voting label fusion amongst the five expert raters. Individual Dice overlaps are shown in Table 4. We detect differences in performance in 5 of the 8 cases (cases 1, 2, 4, 5 and 8), although these differences are observed mainly for haematoma. The most apparent discrepancy is observed in case 5, where the extent of the *MALP-EM* map in the cortical area close to the haematoma is larger (see Fig. 13), allowing the “leakage” of the T2* GRE region of interest. This subsequently led to a wrong FLAIR connected component being selected as haematoma. We also observe T2* GRE “leakage” in case 8, producing a substantial difference in haematoma Dice score. For cases 1 and 2, the disparity in Dice overlaps is due to the re-labelled *MALP-EM* maps having non-ventricular labels for voxels corresponding to intraventricular haemorrhage, resulting in over-segmentation of the haematoma (see Fig. 14). Finally, the overlap difference of case 4 is due to voxels in the haematoma being incorrectly classified as being part of the fourth-ventricle in the proposed registration-based label map, producing haematoma under-segmentation (see Fig. 15).

**Table 4:** Dice overlaps with respect to the label-fusion majority voting amongst the five raters for segmentations performed using T1 MALP-EM maps re-labelled with “lesion” labels and the proposed registration-based labels in 8 cases of Dataset A.

Case	With re-labelled T1 MALP-EM map		With proposed registration-based map	
	Haematoma	Oedema	Haematoma	Oedema
1	0.772	0.821	0.910	0.849
2	0.796	0.766	0.849	0.788
3	0.852	0.767	0.857	0.773
4	0.927	0.870	0.893	0.889
5	0.000	0.000	0.776	0.848
6	0.843	0.899	0.847	0.889
7	0.955	0.931	0.953	0.932
8	0.834	0.808	0.937	0.813

### 3.4 Computing time

We study the computational cost of the proposed algorithm by running 25 repetitions per subject on 7 cases spanning a variety of volume sizes. We run these experiments using MATLAB R2017b on a Windows 7 desktop PC with an Intel^®^ Core™ i7-4820K CPU running at 3.7 GHz and 16 GB of RAM. Results are summarised on Table 5. We observe that the proposed algorithm has very mild computational requirements, and the ability to perform segmentations of sequences with tens of millions of voxels in about a minute or less.

**Table 5:** Mean and standard deviation (in seconds) of running time over 25 repetitions for 7 different subjects. Sequence loading time is the time spent loading all required Nifti files, and running time corresponds to the time spent performing the segmentation.

Sequence loading time		Running time		
Mean (sec.)	Standard dev. (sec.)	Mean (sec.)	Standard dev. (sec.)	Volume size (vox.)
6.6	0.4	6.2	0.2	182 × 224 × 192
9.8	0.6	8.1	0.2	256 × 256 × 160
11.8	0.7	10.5	0.2	256 × 256 × 216
13.9	0.6	12.1	0.1	256 × 256 × 252
32.1	1.0	26.5	0.1	432 × 512 × 160
31.0	1.1	23.6	0.1	512 × 512 × 120
40.3	2.2	36.1	0.1	512 × 512 × 192

## 4 Discussion

In this paper, we have proposed a training-free, unsupervised fully automated algorithm for segmentation of haematoma and perihaematoma oedema from MR images of patients with acute and early subacute spontaneous intracerebral haemorrhage. Results on 2 datasets of 18 and 32 subjects suggest that the proposed method is suitable for reliable segmentation directly from these MR images. We also made the source code of the method publicly available.

One of the main challenges regarding the evaluation of automated segmentation of haematoma and oedema is that there is no gold-standard. This means that there is potential for an important amount of variability between expert raters. This variability is more clearly observed in regions where there is a biological boundary that is not conspicuous in MR. Examples of these boundaries are the interfaces between oedema and white matter lesions, and between haematoma and CSF. Hence, expert rater delineations can only be considered as bronze- or silver-standard in the best case. In the proposed method, we aim to tackle this non-apparent nature of oedema-leukoaraiosis and haematoma-CSF borders by means of the voxel-wise threshold map and the ventricular and WM-GM masks, respectively.

The lack of gold-standard segmentations means that obtaining reliable data to train learning-based algorithms such as *DeepMedic* is not simple. And even if there is enough training data, state-of-the-art algorithms based on FCNN usually require the availability of graphics processing units to train and test. Therefore, the proposed algorithm offers an alternative that does not require training and can be run on a standard CPU to perform haemorrhage and oedema segmentation of SICH patients in about a minute or less, with comparable segmentation performance.

The proposed method comes with two main limitations. Firstly, the *MALP-EM* label maps are propagated from an age-specific template to the individual ICH patients using non-rigid registration, but the accuracy of the registration is limited by missing correspondences introduced by the presence of haematoma and oedema. To minimise the influence of this issue, we utilise a robust similarity measure designed to be less sensitive to these missing correspondences, but there is no guarantee that the resulting labelling will be completely accurate. Secondly, the nature of MR intensities in T2* GRE and FLAIR during the acute and early subacute phase is variable, especially as deoxyhaemoglobin in the ICH core is gradually converted into intracellular methaemoglobin. This could, in some cases, affect the accuracy of the proposed method to quantify haematoma and, consequently, perihaematomal oedema.

Additionally, despite the use of a robust B-Spline interpolation when resampling the T2* GRE and FLAIR images into their corresponding T1 space, partial volume effects are not completely removed. As a consequence, there is a systematic under-segmentation of perihaematoma oedema when located in regions close to CSF. This can also be problematic in clinical settings where the different input images are in mixed planes. Possible solutions are the use of more advanced interpolation techniques, partial volume correction methods, or intensity weight maps based on the distance of WM-GM voxels to the nearest CSF voxel. All these options are potential avenues of future work.

Finally, the Dice scores we obtain for oedema delineation are not as favourable as the ones for haematoma. This may be explained by several reasons. Firstly, the aforementioned partial volume effects have an important impact in the estimation of oedema extent close to CSF. Secondly, oedema segmentation performance strongly depends on how well the haematoma is segmented, since both segmentations share a common border. Therefore, any mistake in haemorrhage segmentation will have an impact on the delineation of oedema, but not vice versa. Lastly, there are cases where the boundary between oedema and leukoaraiosis is not clear, producing disagreement amongst expert raters.

## 5 Conclusion

We have performed a detailed evaluation of the proposed method. Promising results on two datasets demonstrate that our algorithm has the potential to be a useful pre-processing tool for subsequent volumetric assessment of SICH using MRI data. This will allow us to obtain accurate haematoma and perihaematoma oedema definitions to better inform the outcomes of the TICH-2 MRI sub-study. In particular, we will use the resulting automated annotations as regions of interest for evaluation of diffusion characteristics and SICH-related tissue damage of sub-study patients suffering from SICH. Furthermore, with the increasing use of MRI clinically after intracerebral haemorrhage this technique has the potential to inform clinical practice in the future.

## Conflicts of interest

None declared.

## Footnotes

1 http://github.com/BioMedIA/MIRTK.

2 http://www.itksnap.org.

3 Since we have an even number of patients, we take from the two possible median cases the one for which the Dice score is the lowest (i.e. the 10th in ranking of descending order).
