# Supplementary material for: Automated segmentation of haematoma and perihaematomal oedema in MRI of acute spontaneous intracerebral haemorrhage
Source: Comput Biol Med. 2019 Mar;106:126–39. doi: 10.1016/j.compbiomed.2019.01.022 (PMC6382492; doi:10.1016/j.compbiomed.2019.01.022)
Supplement: Supplementary Material [file mmc1.docx]

# Supplementary material

## Effect of λ value on perihaematoma oedema segmentation


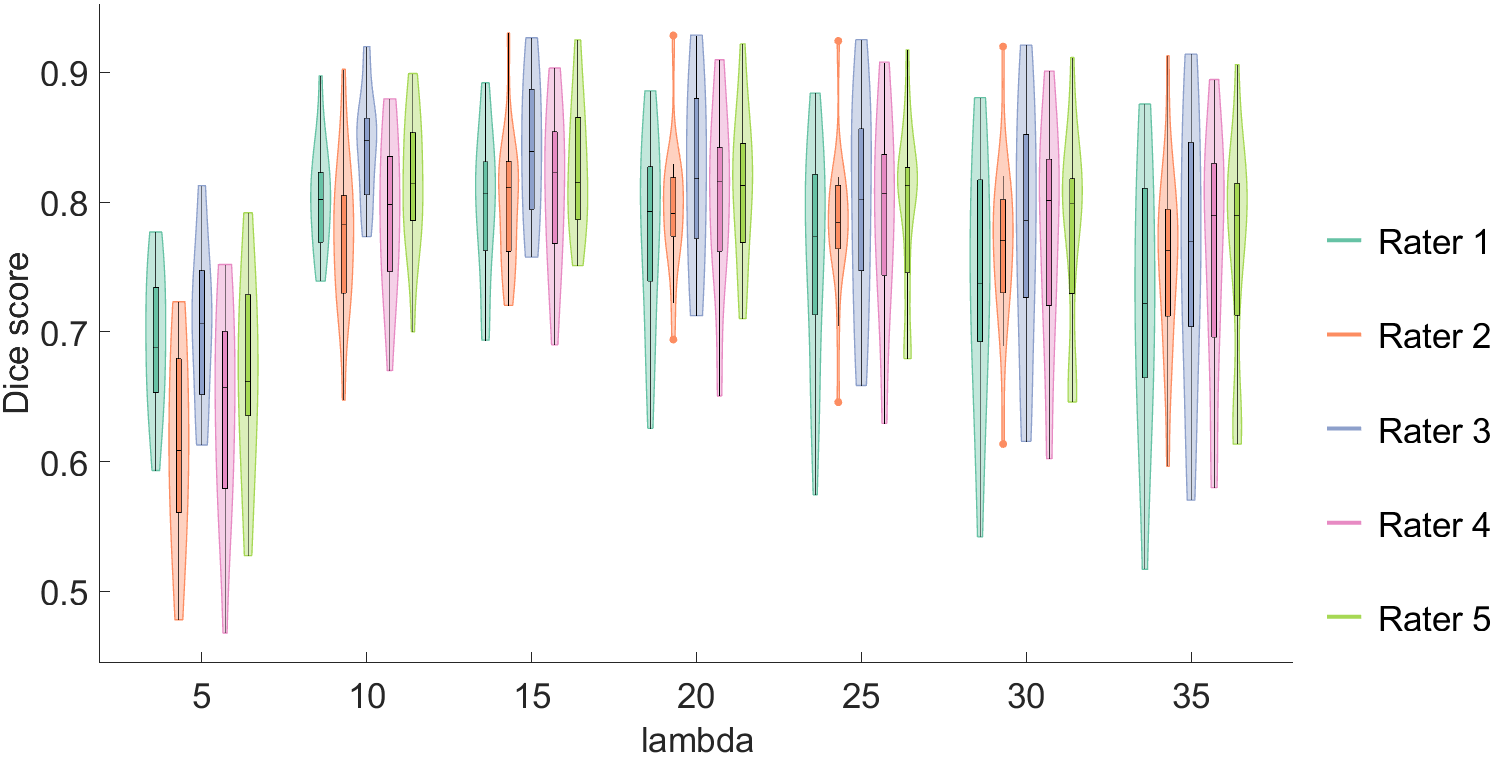


*Figure 1: Box-and-violin plot of Dice scores for different values of the λ parameter.*

We assess the influence of the $\lambda$ parameter on the Dice scores for oedema segmentation. The results are shown in Figure 1. We observe that the best value for this parameter lies around 15, with a sharp deterioration of overlaps for lower values, and a more gradual decrease for higher ones.


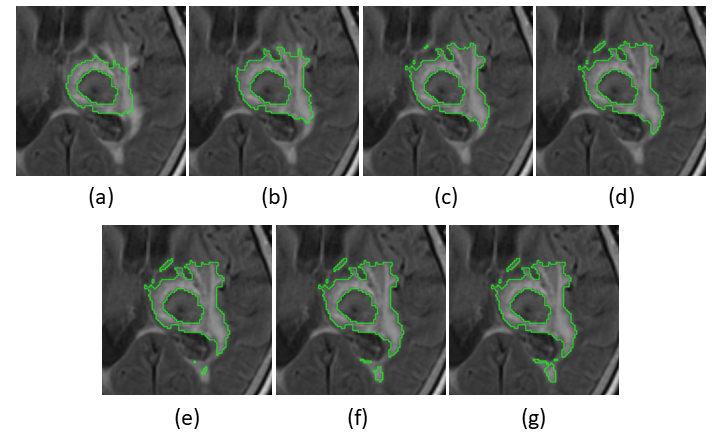


*Figure 2: Visual illustration of the effect of the λ parameter in oedema inclusion. (a) λ = 5. (b) λ = 10. (c) λ = 15. (d) λ = 20. (e) λ = 25. (f) λ = 30. (g) λ = 35.*

We also illustrate how the $\lambda$ parameter affects the inclusion of hyper-intensities further away from the haematoma. From the equation defining the voxel-wise threshold map of hyper-intensities, we can conclude that the greater the value of $\lambda$, the more gradual is the increase of threshold values as a function of the distance to the haematoma. This means that more hyper-intensities are included in the segmentation. This phenomenon is depicted in Figure 2.

## Challenging cases on Dataset B

We present challenging cases on Dataset B where either the proposed algorithm or DeepMedic (or both) tend to produce unsatisfactory results. Figure 3 shows a case where there is over-segmentation of haematoma, for the same reasons as the worst case of Dataset A. A challenging example is shown in Figure 4, where DeepMedic grossly underestimates the extent of haematoma, and the proposed algorithm tends to mistakenly include intraventricular haemorrhage into the segmentation. Lastly, Figure 5 depicts a case where the proposed algorithm performs well, but there is substantial under-segmentation in the DeepMedic result. We believe that an insufficient number of training subjects may be the cause of the underestimations yielded by DeepMedic.


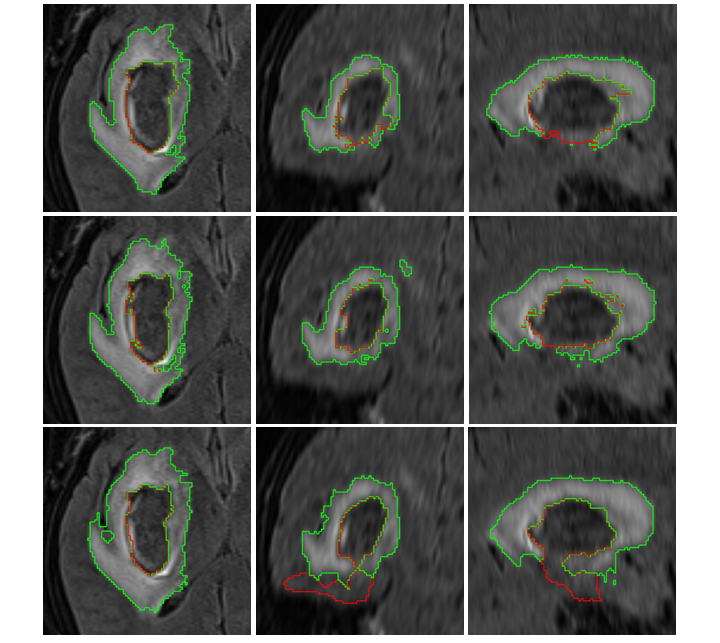


*Figure 3: Axial, coronal and sagittal views of a failure case for the proposed technique. Top row: Manual segmentation. Middle row: DeepMedic. Bottom row: Proposed approach.*


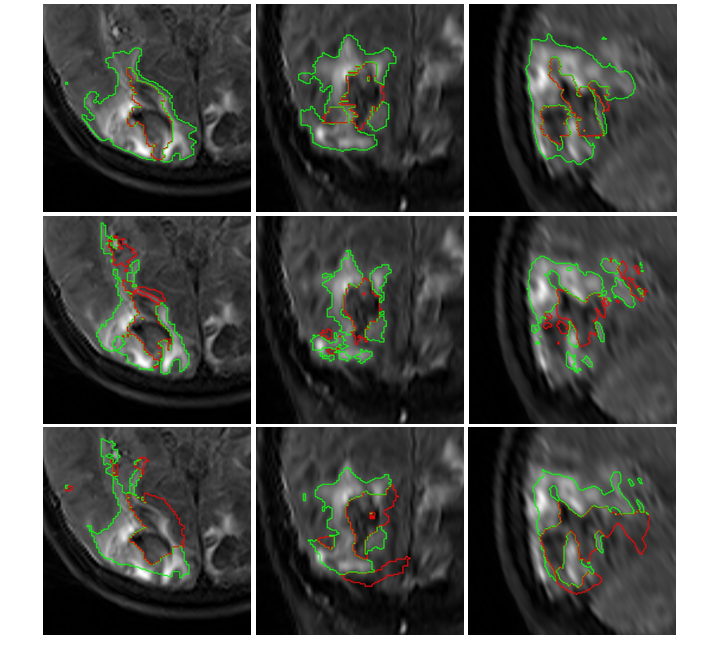


*Figure 4: Axial, coronal and sagittal views of a failure case for both algorithms. Top row: Manual segmentation. Middle row: DeepMedic. Bottom row: Proposed approach.*


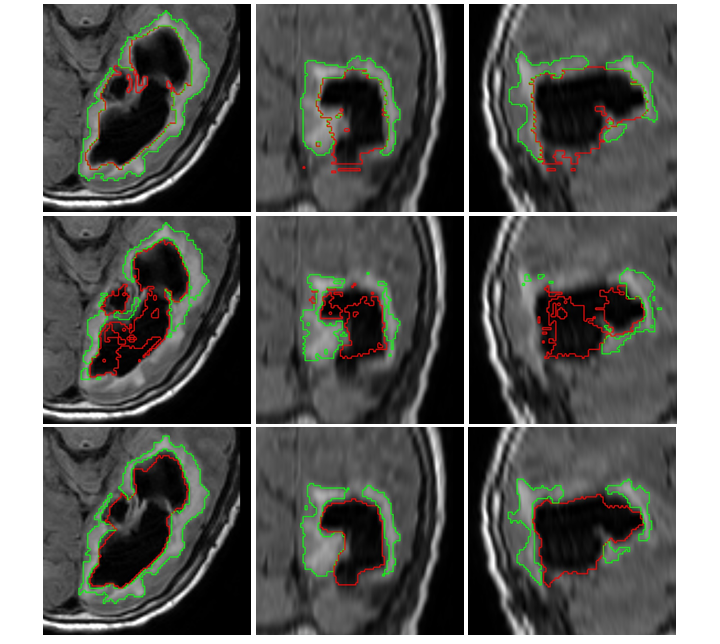


*Figure 5: Axial, coronal and sagittal views of a failure case for DeepMedic. Top row: Manual segmentation. Middle row: DeepMedic. Bottom row: Proposed approach.*

## MR Acquisition parameters

MRI sub-study acquisition parameters for each sequence type are given in the table below. Repetition time (TR), echo time (TE) and inversion time (TI) are measured in milliseconds. Flip angle is measured in degrees. Acquisition matrix, field of view (FOV), slice thickness and slice gap are measured in millimetres.

|  | **T1** | **T2* GRE** | **FLAIR** |
| --- | --- | --- | --- |
| **Dimensionality** | 3D | 2D | 2D |
| **TR** | Minimum | 300-1030 | $\geq$ 11000 |
| **TE** | Minimum | 15-40 | 120-140 |
| **TI** | 450-1100 | – | 2545-2890 |
| **Flip angle** | 8-15 | 15-30 | $\geq$ 90 |
| **Acquisition matrix** | 128-256 | 180-290 | 180-512 |
| **FOV** | 224-256 | 230-240 | 230-240 |
| **Slice thickness** | $\leq$ 1.25 | $\leq$ 4.0 | $\leq$ 4.0 |
| **Slice gap** | – | $\leq$ 1.0 | $\leq$ 0.4 |
| **Number of slices** | 128-256 | 30-50 | 30-42 |

## Scanners

A summary of the different scanners utilised to acquire the sequences is detailed in the table below.

| **Manufacturer** | **Model** | **Field strength** |
| --- | --- | --- |
| General Electric Medical Systems | Discovery MR450 | 1.5T |
| General Electric Medical Systems | Signa HDxt | 1.5T |
| Philips Medical Systems | Achieva | 3T |
| Philips Medical Systems | Ingenia | 1.5T |
| Siemens | Aera | 1.5T |
| Siemens | Avanto | 1.5T |
| Siemens | Prisma | 3T |
| Siemens | TrioTim | 3T |

## Rater expertise

All raters are qualified medical practitioners. Rater 1 has an MSc in translational neuroimaging, Ph.D. in radiological sciences, and 5 years’ experience in cerebrovascular lesion segmentation. Rater 2 is a stroke neurologist with 4 years’ experience in neuroimaging. Raters 3, 4 and 5 are fully-certified consultant clinical neuroradiologists and TICH-2 trial image adjudicators.
